# Patterns and predictors of post-traumatic growth and fear of disease progression in breast cancer patients: a latent profile analysis

**DOI:** 10.3389/fpsyt.2025.1604787

**Published:** 2025-09-18

**Authors:** Keying Guo, Haipeng Li, Weina Du, Ling Cheng, Wei Wang, Zhongtao Zhou, Jing Zhang

**Affiliations:** ^1^ College of Nursing, Bengbu Medical University, Bengbu, Anhui, China; ^2^ College of Mental Health, Bengbu Medical University, Bengbu, Anhui, China

**Keywords:** breast cancer patients, post-traumatic growth, fear of disease progression, latent profile analysis, positive psychology

## Abstract

**Purpose:**

The primary aim of this study is to explore distinct patterns of post-traumatic growth (PTG) and fear of cancer progression (FOP) among breast cancer patients through latent profile analysis (LPA). Additionally, we assessed the differences in demographic and disease-related factors among breast cancer patients with varying patterns. Finally, we examined the influence of socio-demographic, disease-related, social support, anxiety, depression, and post-traumatic stress disorder (PTSD) factors on the varying patterns, aiming to assist healthcare providers in developing more effective psychological care strategies for breast cancer patients.

**Method:**

A questionnaire survey was conducted on 752 breast cancer patients. Latent profile analysis was employed to explore the patterns of post-traumatic growth and fear of cancer progression in these patients, and multiple logistic regression analysis was used to identify the predictive factors for the different patterns.

**Results:**

Based on the fit indices of latent class analysis, a three-class model was identified as the optimal solution, which included the Resisting group, Struggling group, and Growth group. In the Resisting group (24.33%), patients reported low levels of post-traumatic growth and high levels of fear of cancer progression; in the Struggling group (46.14%), patients exhibited moderate levels of post-traumatic growth and low levels of fear of cancer progression; in the Growth group (29.52%), patients demonstrated high levels of post-traumatic growth and moderate levels of fear of cancer progression. Additionally, the multiple logistic regression analysis reveals that marital status, place of residence, education level, disease stage, social support, anxiety, and post-traumatic stress disorder levels in breast cancer patients serve as significant factors influencing the distinct patterns of post-traumatic growth and fear of progression.

**Conclusions:**

This study suggests that there is heterogeneity in the PTG and FOP patterns in breast cancer patients. It provides a research basis for promoting the psychological recovery of breast cancer patients and highlights the importance of focusing on the positive effects of PTG while mitigating the negative impact of FOP. Healthcare providers can implement targeted nursing interventions based on the different patterns observed in breast cancer patients.

## Introduction

Breast cancer is the most prevalent malignancy among women globally, with an estimated 19.96 million new cancer diagnoses in 2022. Of these, 24.1% were reported in China. Furthermore, breast cancer remains the leading cause of cancer incidence among women in China (1). According to the 2024 report from the National Cancer Center of China, breast cancer ranks as the second most common cancer among Chinese women and is the leading cause of cancer incidence among women worldwide. Each year, approximately 429,000 new cases of breast cancer are diagnosed among women in China, accounting for 19.6% of all newly diagnosed cancer cases in women (2). Due to the widespread implementation of breast cancer screening and advancements in treatment technologies, the 5-year survival rate for women with breast cancer in China has increased over the past decade, reaching 80.9%. This reflects an extension in the lifespan of breast cancer patients (3). While physical wounds can be healed through medical treatment, psychological trauma is often harder to overcome. Therefore, the psychological state of patients remains crucial, especially when diagnosed with cancer, which inevitably triggers a series of negative psychological reactions such as anxiety, depression, and concern over disease progression. However, during cancer treatment, positive outcomes may also emerge, such as post-traumatic growth (PTG) (4). PTG refers to the positive psychological changes and transformations that may occur following trauma. Through a process of cognitive integration, individuals can re-evaluate interpersonal relationships, belief systems, attitudes towards life and the future, priorities, and personal strength (5). It aids survivors in finding new meaning, altering lifestyles, and adopting positive behaviors (6), while enabling individuals to reframe traumatic events constructively (7). This is one of how post-traumatic growth can effectively contribute to life extension. Therefore, early detection and enhancement of PTG levels in breast cancer patients are of significant importance in promoting both their physical and psychological recovery.

However, patients exhibiting low levels of PTG may experience negative psychological outcomes, including a fear of cancer progression (FOP). FOP refers to a psychological state in which patients experience fear or concern about the recurrence or progression of a disease. Dysfunctional fear of cancer progression can lead to the development of negative psychological conditions such as depression and anxiety in patients (8, 9). Studies show that fear of cancer progression is a common psychological response among cancer patients and one of the psychosocial needs of cancer survivors (10). At the normal level of fear, patients remain vigilant about their condition, which helps them adapt well and cooperate with treatment, promoting recovery. However, excessive long-term fear can negatively affect disease coping and reduce social functioning and quality of life (11, 12). Previous studies have indicated that the levels of PTG in cancer patients are closely linked to the presence of FOP, exhibiting a negative correlation (13, 14). However, the study by Gu et al. demonstrated a relatively weak association between FOP and PTG (15). Therefore, the potential heterogeneity between PTG and FOP when both coexist remains unclear, suggesting that further exploration of their underlying association is warranted.

Previous studies have extensively explored the factors influencing PTG or FOP, yet there has been limited investigation into the factors affecting the combined patterns of PTG and FOP (16, 17). Current research on PTG in breast cancer patients predominantly adopts a variable-centered approach, treating patients as homogeneous entities and overlooking the inherent heterogeneity between individuals, which results in a lack of personalized care for clinical patients (18). Latent Profile Analysis (LPA) is a statistical technique that categorizes individuals into distinct groups based on objective adaptation indicators, thereby exploring the heterogeneity within populations with similar characteristics (19), which holds significant implications. Therefore, LPA can be utilized as a tool to identify high-risk individuals for implementing tailored intervention strategies (20), assisting in the exploration of different patterns of PTG and FOP in breast cancer patients.

The Theory of Unpleasant Symptoms (TOUS) posits that physiological, psychological, and environmental factors influence an individual’s development. It consists of three main components: symptoms, symptom influencers, and symptom outcomes. The factors affecting symptoms primarily include physiological, psychological, and environmental aspects. These three factors are interrelated and mutually influence each other, collectively associated with the manifestation of symptoms (21). Specifically, variations in PTG and FOP among patients with mastopathy may be shaped by a combination of physiological factors (e.g., disease characteristics), psychological factors (e.g., anxiety and depression levels), and environmental factors (e.g., sociodemographic traits and perceived social support). Concerning physiological factors, previous studies have indicated that disease-related variables, such as the stage of illness, significantly influence the levels of PTG and FOP in patients (22). In terms of psychological factors, a study by Mell et al. demonstrated that levels of anxiety and depression have a significant impact on PTG and FOP in patients with gynecological tumors (23). Regarding environmental factors, Chen et al. found that patients with higher levels of education, better family functioning, and stronger social support exhibited higher levels of PTG, which contributed to improved psychological recovery and lower levels of FOP (24). The choice of TOUS is due to its ability to not only address the occurrence of individual symptoms but also systematically consider the interrelationships between symptoms and their influencing factors. This framework is particularly suitable for exploring the factors influencing different patterns of PTG and FOP in breast cancer patients, as their symptoms typically involve multiple aspects, including physiological, psychological, and environmental factors. Therefore, using TOUS to deeply identify the factors influencing different patterns of PTG and FOP in breast cancer patients can help identify high-risk populations and implement preventive strategies. This is of significant importance for optimizing psychological interventions and improving treatment outcomes.

The application of LPA to explore the PTG and FOP patterns in breast cancer patients contributes to: (a) examining the levels of PTG and FOP across different patterns in breast cancer patients; (b) evaluating the demographic and disease-related differences among breast cancer patients in different patterns; and (c) investigating whether factors such as social support, anxiety, depression, and PTSD levels may influence the likelihood of different patterns in breast cancer patients. The research was primarily guided by three key hypotheses. Hypothesis 1: There are three patterns of PTG and FOP in breast cancer patients, namely mild PTG/high FOP, moderate PTG/moderate FOP, and high PTG/low FOP. Hypothesis 2: Marital status, place of residence, educational level, cancer stage, perceived social support, anxiety, depression, and PTSD symptoms are significant predictors of PTG and FOP patterns. Hypothesis 3: The profile characterized by mild PTG and high FOP is expected to be associated with the highest levels of psychological distress. Our research findings may contribute to the development of targeted nursing interventions by healthcare professionals, aimed at promoting both the psychological and physical well-being of breast cancer patients.

## Methods

### Participants

This cross-sectional study, conducted between 25 July and 10 November 2024, involved 785 breast cancer patients hospitalized in several large tertiary hospitals in northern Anhui Province, China. A total of 785 questionnaires were distributed using a non-random sampling method. Of these, 752 valid responses were received, resulting in a valid response rate of 95.8%. The criteria for inclusion in this study were: 1) Pathological diagnosis of breast cancer; 2) Age ≥ 18 years; 3) No history of other malignancies or prior treatments; 4) Alert and able to communicate effectively without any communication impairments. Exclusion criteria included: 1) Recent use of sedative medications or the presence of metastatic cancer; 2) Co-existing severe cardiac, hepatic, or renal dysfunction; 3) Impaired hearing or speech capabilities that would preclude participation in the study. This research obtained approval from the Ethics Committee of Bengbu Medical University (Approval No. 2024-279), and all participants gave written informed consent.

### Data collecting method

Before completing the questionnaire, trained members of the research team provided standardized instructions to participants, explaining the objectives and significance of the study. Patient data on social determinants and biological-behavioral factors were retrieved from the electronic medical records. Inpatients completed the questionnaire on-site, and the completed forms were immediately collected. An on-site review was conducted to identify any missing responses, and participants were prompted to provide any omitted information without delay. Following the completion of questionnaire collection, a dual verification process was conducted by two researchers. This process adhered strictly to the inclusion and exclusion criteria, and appropriate follow-up questions were posed to clarify any ambiguous statements provided by the patients. Any errors or omissions were promptly corrected and supplemented. After all the questionnaires have been collected, they will undergo a dual verification process by two individuals to eliminate invalid responses, thereby ensuring the authenticity and accuracy of the research data.

### Measures

#### Sociodemographic and disease-related variables

The design was developed through a literature review by the researchers and consultation with clinical nursing experts, and it consists of two sections: demographic information and disease-related data. The demographic data encompasses variables such as age, marital status, educational level, monthly income per capita, Occupied zone, engagement in activities (e.g., reading, painting) within the past month, physical exercise, occupation, and average nightly sleep duration of cancer patients. The disease-related data includes factors such as whether chemotherapy was administered and the cancer stage, and the disease stage is classified as I to IV.

#### Post-traumatic growth scale

This tool was originally developed by Tedeschi and Calhoun, and subsequently adapted by Chinese scholar Wang Ji (25). The scale has been widely applied in studies involving 27 distinct trauma populations across China. It includes five aspects: life perception, personal strength, new possibilities, interpersonal relationships, and self-transformation, comprising a total of 20 items. The scale uses a 6-point Likert scoring system, with each item rated from “not at all” to “very much,” which translates to scores of 0 through 5. The cumulative score can vary between 0 and 100, with elevated scores reflecting a higher level of post-traumatic growth. In the present research, the Cronbach’s α for this scale was found to be 0.959.

#### Fear of disease progression scale

The scale was initially developed by Mehnert et al. (26), and subsequently adapted into Chinese by Wu Qiyun et al. (27). It is primarily utilized for quantifying patients’ fear regarding the progression of their disease. It encompasses two dimensions: physical health, and social and family functions, with a total of 12 items. Using a 5-level Likert scoring method, each item offers five options ranging from “never” to “always,” corresponding to scores from 1 to 5. The total score ranges from 12 to 60 points, with higher scores signifying a greater fear of disease progression. The Cronbach’s α coefficient of the scale in this study was 0.835.

#### Social support rating scale

This research employed the Social Support Rating Scale (SSRS) created by Xiao Shuiyuan in 1986 (28). The scale outlines three aspects: objective support, subjective support, and social support use, comprising a total of 10 items. The total score of social support is obtained by summing the scores of all items, with a range from 12 to 66. Higher scores reflect greater social support. The Cronbach’s α for this scale in the present study was 0.895.

#### Hospital anxiety and depression scale

The Hospital Anxiety and Depression Scale (HADS), developed by Zigmond et al. in 1983, is a self-report scale used to screen for anxiety and depression in hospitalized patients (29). The HADS includes 14 items, split into two subscales: anxiety and depression, each containing 7 items. Each item is rated on a 4-point Likert scale, offering four response choices that range from 0 to 3, with elevated scores reflecting greater symptom severity. In this study, Cronbach’s value for the scale was found to be 0.865.

#### Post-traumatic stress disorder scale

The measurement tool was created by the Behavioral Science Department at the U.S. National Center for PTSD, grounded in the DSM-IV criteria (30). It was subsequently updated by Jiang Chao and colleagues for application within the Chinese demographic (26). It is an effective screening tool for post-traumatic stress disorder. The scale comprises 17 items spanning three categories: re-experiencing, avoidance/numbing, and hyperarousal. Each item is scored from 1 to 5, ranging from “not at all” to “extremely,” with a total score range of 17 to 85. Higher scores suggest more pronounced symptoms of PTSD. In this study, Cronbach’s α for this scale was found to be 0.923.

### Statistical methods

This study utilized SPSS 27.0 and Mplus 8.3 for statistical analysis. To address missing data, the study employed the method of multiple imputation. This approach generates a complete dataset by using model estimation and repeated simulations. Descriptive statistics were computed using mean ± standard deviation for continuous data that met the assumption of normality, and frequencies and percentages for categorical or ordinal data. To maximize the interpretability of various solutions and facilitate model convergence, the five dimensions of the PTG scale and the two dimensions of the FOP scale were employed as indicators in the LPA. Given that PTG employs a 6-point Likert scale and FOP utilizes a 5-point Likert scale, and considering the differences in the scoring ranges of each dimension for both FOP and PTG, the scores for all seven variables were first converted into T-scores to facilitate interpretation. The T-score was calculated as: T = 50 + 10 × Z, where Z = (X − *x*¯)/S (31). The LPA, a person-centered statistical approach, addresses this limitation by identifying latent subgroups using continuous indicator variables (32). LPA was conducted using Mplus, with models fitting 1 to 4 latent classes to determine the best-fitting model. The differences between expected and actual values were compared using the likelihood ratio chi-square test, Akaike Information Criterion (AIC), Bayesian Information Criterion (BIC), and sample size-adjusted BIC (aBIC), with smaller values indicating better model fit. The bootstrap likelihood ratio test (BLRT) and the Lo-Mendell-Rubin likelihood ratio test (LMR) were used to compare the fit differences between models. The entropy value, which is closer to 1, indicates a more precise classification.

The optimal classification model for PTG and FOP patterns in breast cancer patients was determined based on the results of latent profile analysis. A Pearson correlation analysis was conducted to examine the relationship between PTG and FOP. Chi-square tests or one-way ANOVA were performed using SPSS 27.0 to compare the differences in demographic data, disease-related information, social support, anxiety, depression, and post-traumatic stress disorder across different categories. An unordered multinomial logistic regression model was employed, with the latent categories of PTG and FOP patterns in breast cancer patients as the dependent variables. Factors exhibiting statistically significant differences in univariate analyses were used as independent variables to investigate the factors influencing different categories of breast cancer patients, with a significance threshold set at P < 0.05. In the multicollinearity test, this study utilizes the variance inflation factor (VIF) as a criterion for assessing collinearity. Specifically, VIF > 5 indicates the potential existence of multicollinearity among the explanatory variables, whereas VIF < 5 suggests the absence of multicollinearity issues (33).

## Results

### Sociodemographic characteristics

A total of 785 breast cancer patients were enrolled in this study, of which 752 completed questionnaires were deemed valid, corresponding to a validity rate of 95.8%. The demographic characteristics of the patients are summarized in **Table 1**.

**Table 1:** Descriptive statistics for sociodemographic and disease information (n = 752).

Variables	M (SD) or n (%)
Marital status	
married	690(91.8)
unmarried	62(8.2)
Per capita monthly income	
≤2000	333(44.4)
2000-5000	263(34.9)
≥5000	156(20.7)
Educational level	
≤6 years	388(51.5)
>6 yeas	364(48.5)
Occupied zone	
village	422(56.2)
city	330(43.8)
Physical training	
once a week or more	439(58.4)
no exercise	313(41.6)
Have you participated in activities such as reading, painting, etc. in the past month	
yes	262(34.8)
no	490(65.2)
Profession	
low-skilled (waiters, drivers and so on)	534(70.9)
high knowledge occupation (university teacher and so on)	218(29.1)
Sleep every night duration	
≥6h	401(53.3)
<6h	351(46.7)
Chemotherapy	
yes	440(58.4)
no	312(41.6))
Disease staging	
I stage	86(11.4)
II stage	120(15.9)
III stage	452(60.0)
IV stage	94(12.7)
Continuous variable	
Age	54.37(11.2)
HADS-A	8.40(4.2)
HADS-D	8.72(4.1)
PTSD	36.12(12.9)
SSRS	34.24(6.3)

### Correlations between PTG and FOP

The Pearson correlation coefficient between PTG and FOP is shown in **Table 2**. The results indicate a negative correlation between the total PTG score and subscale scores with the total FOP score and subscale scores.

**Table 2:** Correlations between PTG and FOP (n = 752).

Variables	M(SD)	1	1.1	1.2	1.3	1.4	1.5	2	2.1	2.2
1 Post-traumatic growth	47.67(18.94)	1								
1.1 Spiritual transformation	9.45(3.86)	.932**	1							
1.2 Appreciation of life	13.65(5.78)	.939**	.868**	1						
1.3 Relationship with others	7.27(3.48)	.889**	.783**	.795**	1					
1.4 Personal strength	7.64(3.24)	.883**	.788**	.769**	.774**	1				
1.5 New possibilities	9.65(4.46)	.893**	.788**	.769**	.733**	.752**	1			
2 Fear of disease progression	33.44(9.53)	-.170**	-.179**	-.158**	-.096**	-.131**	-.190**	1		
2.1 Physical health fear	16.67(5.35)	-.159**	-.162**	-.142**	-.105**	-.125**	-.177**	.906**	1	
2.2 Social family fear	16.77(5.20)	-.149**	-.163**	-.146**	.069	-.114**	-.167**	.900**	.631**	1

M, mean; SD, standard deviation.

**: at the 0.01 level (two-tailed), the correlation is significant.

### Fit index of the LPA

**Figure 1:**
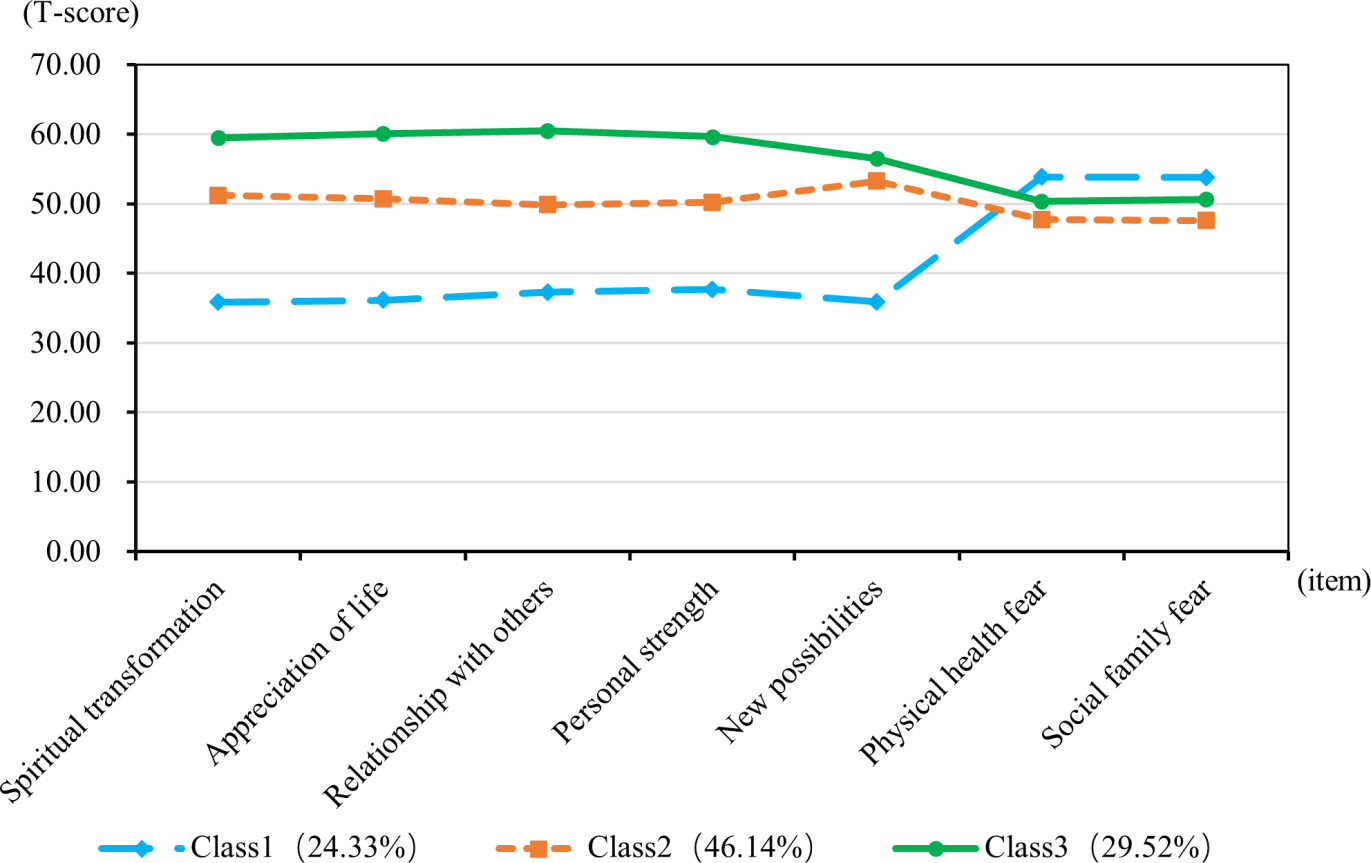
T-scores for subscales of post-traumatic growth and fear of disease progression for the three-class latent profile model.

A latent profile analysis was conducted for 752 breast cancer patients, focusing on the PTG and FOP model scores, with the 7 dimension scores serving as manifest indicators. Latent profile models with 1 to 4 classes were sequentially fitted starting from the baseline model of class 1, as detailed in **Table 3**. As the number of categories increased, the AIC, BIC, and aBIC all decreased. However, when fitting the model with 3 classes, the AIC, BIC, and aBIC showed a flattening trend. The P-value of the LMRT was <0.001, and the Entropy was 0.916, which is greater than 0.900. Considering both the model fit indices and the practical significance of the classification, Model 3 was selected as the best fitting model. The latent profile plot for Model 3 is shown in **Figure 1**.

**Table 3:** Fit indexes for LPA models.

Class	AIC	BIC	aBIC	Entropy	P_LMR_	P_BLMR_	Proportion
1	39198.311	39263.029	39218.574				
2	36573.802	36675.502	36605.643	0.966	<0.001	<0.001	0.265,0.735
3	35758.989	35897.671	35802.409	0.916	<0.001	<0.001	0.243,0.461,0.295
4	35407.782	35583.446	35462.781	0.897	<0.001	<0.001	0.178,0.345,0.197,0.279

AIC, Akaike information criterion; BIC, Bayesian information criterion; aBIC, Sample-adjusted BIC; LMRT, Lo-Mendell-Rubin test; BLRT, Bootstrap likelihood ratio test.

### Patterns of PTG and FOP among patients

**Figure 2:**
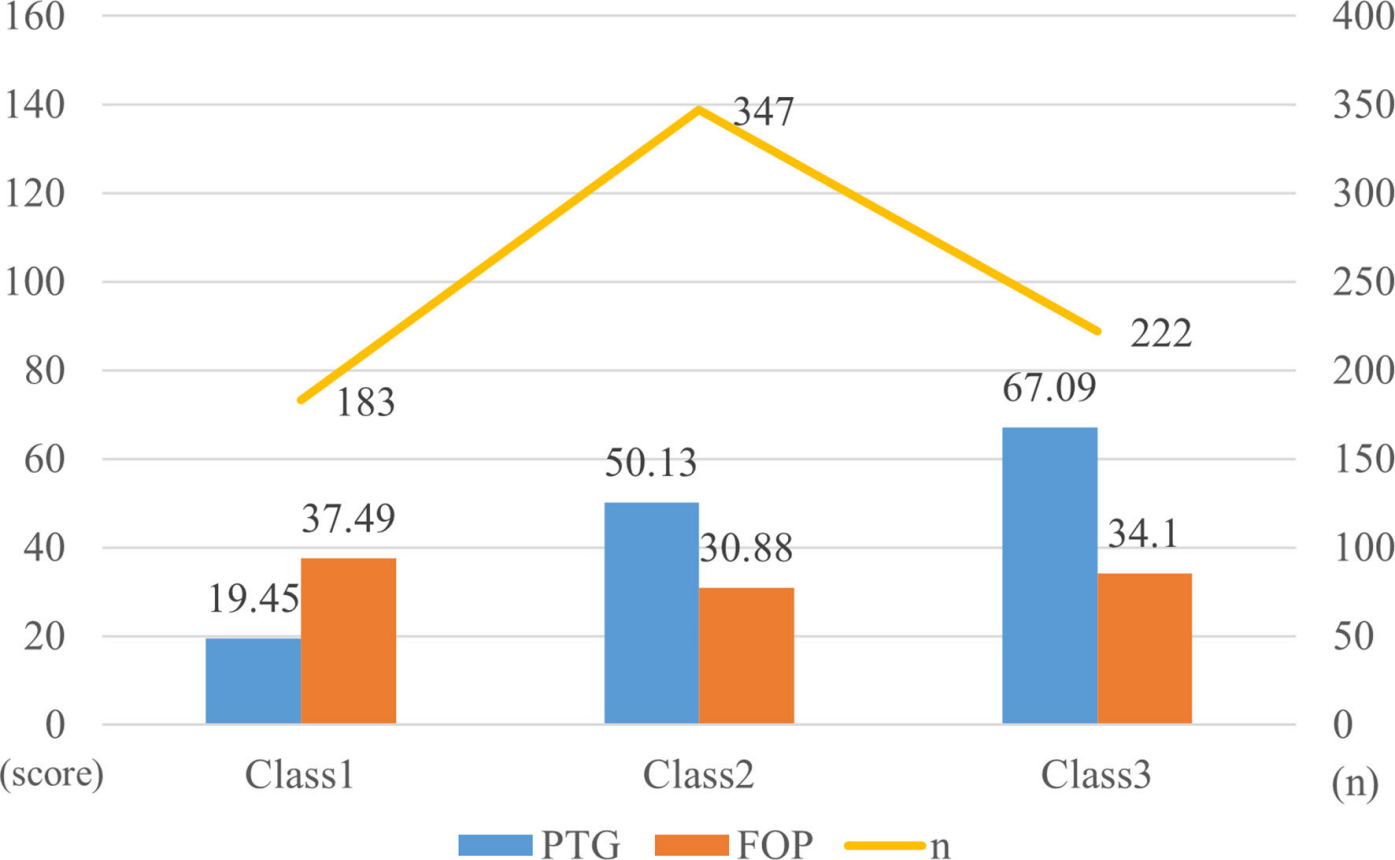
Parameters for the three class patterns.

The T-score subscales for PTG and FOP in three distinct modes are shown in **Figure 1**. The scores for the three categories (PTG and FOP) are presented in **Figure 2**. In model 1, patients exhibit lower PTG scores and higher FOP scores, indicating that this group struggles to find positive meaning in their trauma and lacks confidence in their recovery. Consequently, this group is labeled as the “Resisting Group.” This group consists of 183 patients, representing 24.33% of the total sample. In model 2, patients’ PTG scores fall between Categories 1 and 3, indicating moderate PTG, while FOP scores are lower. This suggests that these patients are able to face their condition more rationally, but experience limited post-traumatic growth and remain in a state of ongoing effort and psychological struggle. Therefore, this group is labeled the “Struggling Group,” comprising 347 patients, which accounts for 46.14% of the total sample. In model 3, patients’ scores across the five dimensions of PTG were higher than those in models 1 and 2, with PTG reaching its highest level, whereas FOP scores ranged between models 1 and 3. This indicates that these individuals are capable of achieving personal growth and positive psychological transformation following trauma. Accordingly, this group is labeled the “Growth Group,” consisting of 222 patients, accounting for 29.52% of the total sample.

### Differences in sociodemographic factors, disease-related factors, social support, anxiety and depression, and post-traumatic stress disorder in the three patterns

The results of the univariate analysis indicated that significant differences were observed across the three groups in terms of patient age, marital status, education level, living area, recent cognitive activities, occupation, sleep duration, chemotherapy status, disease stage, anxiety, depression, social support, and post-traumatic stress disorder (P<0.005), as shown in **Table 4**.

**Table 4:** Univariate analysis of three different patterns (N = 752).

Variables	Resisting group n/M (SD)	Struggling group n/M (SD)	Growth group n/M (SD)	χ^2^/F	*P*
Marital status					
married	173(94.5)	325(93.7)	192(86.5)	11.680^1)^	0.003
unmarried	10(5.5)	22(6.3)	30(13.5)		
Per capita monthly income					
≤2000	95(51.9)	146(42.1)	92(41.4)	9.408^1)^	0.052
2000-5000	63(34.4)	119(34.3)	81(36.5)		
≥5000	25(13.7)	82(23.6)	49(22.1)		
Educational level					
≤6 years	128(69.9)	155(44.7)	105(47.3)	32.982^1)^	<0.001
>6 years	55(30.1)	192(55.3)	117(52.7)		
Occupied zone					
village	132(72.1)	174(50.1)	116(52.3)	25.431^1)^	<0.001
city	51(27.9)	173(49.9)	106(47.7)		
Physical training					
once a week or more	95(51.9)	210(60.5)	134(60.4)	4.162^1)^	0.125
no exercise	88(48.1)	137(39.5)	88(39.6)		
Have you participated in activities such as reading, painting, etc. in the past month					
yes	45(24.6)	125(36.0)	92(41.4)	12.944^1)^	0.002
no	138(75.4)	222(64.0)	130(58.6)		
Profession					
Low-skilled occupation (waiters, drivers)	144(78.7)	229(66.0)	161(72.5)	9.729^1)^	0.008
High knowledge occupation (university teacher)	39(21.3)	118(34.0)	61(27.5)		
Sleep every night duration					
≥6h	62(33.9)	221(63.7)	118(53.2)	42.778^1)^	<0.001
<6h	121(66.1)	126(36.3)	104(46.8)		
Chemotherapy					
Yes	125(68.3)	206(59.4)	109(49.1)	15.438^1)^	<0.001
No	58(31.7)	141(40.6)	113(50.9)		
Disease staging					
I stage	20(10.9)	34(9.8)	32(14.4)	32.241^1)^	<0.001
II stage	33(18.0)	50(14.4)	37(16.7)		
III stage	89(48.6)	236(68.0)	127(57.2)		
IV stage	41(22.4)	27(7.8)	26(11.7)		
Scale (means ± SD)					
Age	57.07±10.17	53.34±11.21	53.75±11.61	7.250^2)^	<0.001
HADS-A	10.97±3.55	7.25±3.86	8.08±4.38	54.193^2)^	<0.001
HADS-D	10.99±3.78	7.58±3.74	8.65±4.01	47.729^2)^	<0.001
PTSD	45.40±12.77	33.07±10.94	35.24±12.09	75.384^2)^	<0.001
SSRS	30.91±5.26	35.48±6.28	35.04±6.10	37.553^2)^	<0.001

HADS-A refers to the Hospital Anxiety and Depression Scale - Anxiety Subscale, HADS-D refers to the Hospital Anxiety and Depression Scale - Depression Subscale, SSRS refers to the Social Support Rating Scale, and PTSD refers to the Post-Traumatic Stress Disorder Scale.

^1)^ *χ* ^2^ value, ^2)^ *F* value.

Furthermore, a multinomial logistic regression analysis was conducted using the latent classes of PTG and FOP patterns among breast cancer patients as the dependent variable (with the resisting group as the reference category), and the factors found to be statistically significant in the univariate analysis were included as independent variables (**Table 5**). The results showed that, compared to the resisting group, married patients were more likely to belong to the growth group (OR = 3.359, P = 0.006). Patients in stage I (OR = 3.431, P = 0.011; OR = 2.715, P = 0.041) and stage III (OR = 4.788, P<0.001; OR = 2.694, P = 0.003), as well as those with higher social support levels (OR = 1.058, P = 0.011; OR = 1.056, P = 0.019), were more likely to be classified into the struggling group and growth group. In contrast, when compared to the struggling group and growth group, patients more likely to be classified into the resisting group include those with: less than 6 years of education (OR = 0.458, P = 0.003; OR = 0.554, P = 0.020), rural residency (OR = 0.458, P<0.001; OR = 0.428, P<0.001), anxiety (OR = 0.891, P = 0.003; OR = 0.899, P = 0.009), and a higher severity of post-traumatic stress disorder (OR = 0.956, P<0.001; OR = 0.936, P<0.001). The results of the multicollinearity test indicated that all VIF values were less than 5, suggesting that multicollinearity is not a concern (**Supplementary Table 1**).

**Table 5:** Results of multiple logistic regression analysis for three different patterns (n = 752).

Model Variables	Struggling group ^a^			Growth group ^a^		
	OR	P	95%CI	OR	P	95%CI
Marital status(unmarried)						
married	1.778	0.209	0.724-4.365	3.359	0.006	1.406-8.029
Educational level(>6 years)						
≤6 years	0.458	0.003	0.306-0.788	0.554	0.020	0.337-0.911
Occupied zone(city)						
village	0.458	<0.001	0.285-0.735	0.428	<0.001	0.26.-0.704
Disease staging (IV stage)						
I stage	3.431	0.011	1.321-8.912	2.715	0.041	1.043-7.067
II stage	2.064	0.083	0.910-4.681	1.384	0.445	0.601-3.184
III stage	4.788	<0.001	2.492-9.198	2.694	0.003	1.393-5.211
Scale (means ± SD)						
SSRS	1.058	0.011	1.013-1.105	1.056	0.019	1.009-1.106
HADS-A	0.891	0.003	0.826-0.962	0.899	0.009	0.830-0.973
PTSD	0.956	<0.001	0.935-0.978	0.936	<0.001	0.915-0.959

a The reference category is profile 1 (N = 183). OR, odds ratio. 95% CI, 95% confidence interval of OR.

## Discussion

### The characteristics of different patterns of PTG and FOP in breast cancer patients

In this study, we conclude that the three-class model is the most effective and accurate for breast cancer patients exhibiting different patterns of PTG and FOP. In this study, based on the characteristics of each model, three distinct PTG and FOP patterns were named: the Resisting group (Low PTG/High FOP), the Struggling Group (Moderate PTG/Low FOP), and the Growth Group (High PTG/Moderate FOP). These patterns resemble those identified in previous studies concerning post-traumatic stress symptoms and post-traumatic growth in breast cancer patients (34). Based on the TOUS framework, we systematically evaluated the physiological, psychological, and environmental aspects of breast cancer patients. We identified seven influencing factors within the PTG and FOP models: physiological dimension (disease stage), psychological dimension (anxiety and PTSD), and environmental dimension (marital status, residence, anxiety level, and social support). The Resisting group, accounting for 24.33%, is characterized by low PTG/high FOP and represents the most severe pattern among the three. Our study provides a reference for interventions based on different PTG and FOP patterns in breast cancer patients.

Our research findings indicate that 29.52% of breast cancer patients belong to the Growth group, characterized by high levels of PTG and moderate FOP, while 24.33% of patients belong to the Resisting group, characterized by low levels of PTG and high levels of FOP. The underlying cause of this phenomenon could be that individuals who undergo PTG often demonstrate more advanced emotional regulation and coping strategies in response to stress. Through constructive reflection on traumatic experiences, such patients may become more adept at managing and reducing anxiety associated with the potential recurrence of cancer (35). According to the theory of emotional cognitive evaluation (36), individuals must consistently assess and modulate the effects of external stressors on their emotional responses in order to sustain an optimal state of well-being. This self-regulation behavior, through the evaluation of one’s negative emotions and coping strategies, facilitates psychological recovery, enhancing breast cancer patients’ psychological well-being. It fosters the development of a positive mindset, improves PTG levels, and enables patients to confront the traumatic events associated with the disease and its treatment with a constructive attitude, thereby mitigating their FOP. Conversely, when patients exhibit insufficient PTG, the fear of disease progression becomes more pronounced. Moreover, 46.14% of breast cancer patients belong to the Struggling group, characterized by moderate PTG levels and low FOP levels. In this study, breast cancer patients in the struggling group exhibited lower FOP levels, which can be attributed to their stronger self-efficacy. This enhanced self-efficacy encouraged proactive rehabilitation behaviors, leading to a stable post-treatment state and reduced FOP. Additionally, these patients displayed a more optimistic outlook and, through psychological adjustments, achieved moderate PTG (37).

### Predictive factors influencing the PTG and FOP patterns of breast cancer patients

#### Physiological dimension

The study found that patients with stage I and stage III breast cancer were more likely to be categorized into the Struggling group and Growth groups. This phenomenon may be because stage I breast cancer patients, with their better prognosis and higher chances of cure, face fewer physiological and psychological challenges. As a result, they are more able to approach the future positively. This cognitive shift helps patients face future challenges with confidence, draw strength from their trauma, and thus reduce the fear of cancer recurrence (38). On the other hand, patients with stage III breast cancer face more challenges and uncertainties. Due to the disease’s status, they may experience emotional distress, leading to psychological difficulties. In this context, patients can achieve a deeper understanding of life through psychological self-transcendence, which enhances their level of post-traumatic growth (39). Healthcare professionals should guide patients, particularly those with advanced breast cancer, in adopting positive coping strategies, such as psychological counseling and social support networks, to alleviate psychological distress and enhance mental well-being.

#### Psychological dimension

This study found that patients with higher levels of anxiety are more likely to be classified into the Resisting group. Related studies have shown that the anxiety levels of breast cancer patients are closely associated with PTG and FOP. Anxiety is significantly associated with lower levels of PTG in breast cancer patients, potentially reflecting difficulties in trauma processing and reduced likelihood of PTG development. Additionally, higher anxiety levels exacerbate the degree of FOP in these patients (40, 41). The severity of anxiety in patients negatively impacts the development of PTG, while lower levels of anxiety serve as a critical buffer against stress. A strong psychological resilience helps breast cancer patients quickly adapt to and cope with traumatic events, thereby enhancing PTG levels (42), and minimizing the degree of FOP in patients. Healthcare professionals can use psychological interventions to assess the physical and mental health of breast cancer patients. For those with abnormalities, referral to oncology for multidisciplinary treatment can promote PTG and alleviate FOP (43).

Our study also found that patients with a higher degree of post-traumatic stress disorder (PTSD) are more likely to be classified into the Resisting group. Cancer patients often experience trauma-related symptoms, making them more susceptible to developing PTSD (44). Studies have shown that PTSD in breast cancer patients is negatively correlated with PTG and positively correlated with FOP (45, 46). The potential cause of this phenomenon may be that breast cancer diagnosis or multiple traumas intensify PTSD symptoms. These symptoms not only threaten physical health but also negatively affect mental well-being, thereby exacerbating FOP levels and hindering PTG development in breast cancer patients (47). PTSD refers to a set of characteristic and persistent symptoms that occur after an individual experiences an extraordinary traumatic event (48). It can lead to feelings of hopelessness, depression, and suicidal thoughts, significantly impacting both the psychological and physical health of breast cancer patients (49). Therefore, PTSD plays a crucial role in the PTG and FOP levels of breast cancer patients. Therefore, healthcare professionals should consistently monitor and assess the psychological stress levels of breast cancer patients. When necessary, cognitive-affective training interventions can be implemented, which may contribute to enhancing patients’ psychosocial adaptation to the disease, improving their treatment adherence, and promoting recovery (50).

#### Environmental dimension

Our study reveals that married breast cancer patients are more likely to be categorized into the Growth group, indicating that marital status may serve as a predictor of three distinct patterns of PTG and FOP in these patients. The possible cause of this phenomenon may be that, compared to unmarried patients, married breast cancer patients communicate more frequently with their spouses, enabling them to more deeply perceive the support from their partners (51). Research has shown (52, 53) that PTG is a common outcome jointly influenced by both the patient and their spouse. Some married patients, during the process of experiencing trauma related to breast cancer diagnosis, treatment, and recovery, can transform their suffering into something positive, thereby promoting PTG and alleviating the patient’s level of FOP. Unmarried women may experience heightened anxiety regarding the potential impact of a breast cancer diagnosis on their future fertility, which could contribute to an elevation in their FOP levels and consequently result in reduced PTG levels in breast cancer patients. Therefore, healthcare professionals should provide unmarried women with comprehensive health education about breast cancer to alleviate their anxiety. Additionally, they should emphasize the importance of support from family members of unmarried breast cancer patients, strengthen interactions within the family, and offer internal support to the patient. This approach will help promote psychological recovery and foster PTG in unmarried patients (54).

In this study, breast cancer patients with ≤ 6 years of education and those living in rural areas are more likely to be categorized into the Resisting group. Studies suggest that the level of education in breast cancer patients is positively correlated with PTG. Individuals with higher educational attainment tend to approach problems from a more comprehensive perspective and demonstrate a more optimistic attitude toward negative life events. In contrast, patients with lower education levels have fewer cognitive resources to cope with stress and are less able to engage in deeper reflection on traumatic experiences (55).At the same time, lower educational levels are associated with higher levels of FOP in patients. A greater awareness of risk factors and the impact of cancer recurrence may contribute to more catastrophic thinking, which, in turn, intensifies the severity of FOP and leads to a decline in the patients’ level of PTG (56). Research has also shown (38, 57) that rural breast cancer patients have lower PTG scores compared to their urban counterparts. This may be due to the relative lack of medical resources in rural areas and fewer channels for patients to access information about the disease. As a result, these patients have a limited understanding of breast cancer, leading to greater fear of the disease, which deepens their FOP and, consequently, hinders their PTG. Therefore, we recommend that clinical staff provide health education to breast cancer patients with lower educational levels, especially in remote rural areas. This can be achieved by encouraging lifestyle adjustments, fostering a positive mindset, and promoting regular check-ups, enabling patients to better understand their condition, actively participate in treatment decisions, effectively manage their illness, and ultimately improve their overall quality of life and prognosis.

Furthermore, our study further reveals that patients with higher levels of social support are more likely to be categorized into the Struggling group and growth group. Previous studies have demonstrated that social support plays a critical role in fostering PTG (58). Through self-disclosure and the augmentation of social support resources, it can significantly aid breast cancer patients in reintegrating into the workforce while sustaining and enhancing their overall physical and psychological well-being. Social support encompasses various dimensions, which can significantly influence the psychological well-being of breast cancer patients. Emotional support from family, friends, and social networks, in particular, offers comfort and understanding, alleviating feelings of loneliness and anxiety. As a result, it can also help reduce the severity of FOP in these patients to some extent (59, 60). In the study conducted by Tedeschi (30), it is highlighted that social support, particularly that derived from partners, family, or friends, plays a crucial role in enhancing cognitive functions, especially in helping cancer patients to find personal meaning in the face of life-threatening situations and fostering PTG. Therefore, we believe that healthcare professionals should prioritize the impact of social support on breast cancer patients by educating the patients’ families, friends, and significant others on when and how to provide support, including emotional and financial assistance, to help them cope with the disease (61).

### Clinical significance

Firstly, our study integrates PTG and FOP into a unified model for breast cancer patients, aiming to investigate the distinct characteristics of breast cancer patients in different models. This approach highlights the heterogeneity of the condition and offers novel perspectives for clinicians in the context of psychological rehabilitation. Secondly, our findings suggest that clinicians should pay greater attention to breast cancer patients who are unmarried, live in rural areas, have lower levels of education and social support, and exhibit higher levels of anxiety and PTSD. These individuals tend to fall into the Resisting group, which is characterized by low levels of PTG and high levels of FOP, potentially leading to adverse psychological outcomes. Finally, from a treatment perspective, clinicians should consider the distinct patterns of breast cancer patients when developing and tailoring personalized treatments. For example, patients in the Growth group, who exhibit higher levels of PTG, may be better equipped to cope with trauma. Therefore, routine care should suffice for this group without the need for additional psychological interventions. For patients in the Struggling group, moderate social activities can help build a support system, enhance self-efficacy, and reduce FOP levels. In the Resisting group, interventions should prioritize PTG needs, with Cognitive Behavioral Therapy (CBT) as the primary approach (62). This therapy can help patients adjust their mindset and promote PTG. If necessary, medications such as antidepressants or anxiolytics may be considered, but only under professional supervision. In addition, healthcare professionals can support breast cancer patients through professional psychological guidance, helping them gradually rediscover meaning and hope in life following trauma, thereby promoting PTG. Early identification of patients’ FOP levels is also crucial, enabling timely interventions that encourage a more positive psychological outlook toward the disease and support recovery.

### Limitation

There are several limitations to our study. First, this is a cross-sectional study, meaning it does not account for the changes in PTG and FOP over time in breast cancer patients. Second, the sample data were all derived from Anhui Province, which may limit the generalizability of the findings. Finally, our study did not take into account other psychological variables that might influence the results or the patterns of PTG and FOP among patients. In addition, only self-report questionnaires were used. Therefore, future research could involve expanding the sample size and diversity, conducting longitudinal and qualitative studies, and employing latent class growth modeling to explore the causal relationships between PTG, FOP, and longitudinal trajectory categories.

## Conclusion

Our study identified three latent classes of PTG among breast cancer patients: the Resisting group, the Struggling group, and the Growth group. Additionally, our findings indicated that marital status, place of residence, educational level, disease stage, SRSS, anxiety, and PTSD levels were influential factors affecting the patterns of PTG and FOP in breast cancer patients. Patients with different PTG and FOP patterns may have distinct care needs, thus healthcare providers can implement targeted nursing interventions based on the different patterns observed in breast cancer patients.

## Data Availability

The raw data supporting the conclusions of this article will be made available by the authors, without undue reservation.
