# Supplementary material for: Patterns and predictors of post-traumatic growth and fear of disease progression in breast cancer patients: a latent profile analysis
Source: Front Psychiatry. 2025 Sep 18;16:1604787. doi: 10.3389/fpsyt.2025.1604787 (PMC12488680; doi:10.3389/fpsyt.2025.1604787)
Supplement: Supplementary file 1 [file Table1.docx]

**Supplement table1** Multicollinearity test (n = 752)

| Variable | VIF | 1/VIF |
| --- | --- | --- |
| Marital status | 1.081 | 0.925 |
| Educational level | 1.073 | 0.931 |
| Occupied zone | 1.087 | 0.919 |
| Disease staging | 1.069 | 0.935 |
| SSRS | 1.319 | 0.758 |
| HADS-A | 1.505 | 0.664 |
| PTSD | 1.544 | 0.647 |
